# First report of opportunistic screening for nasopharyngeal carcinoma by gastroscopy

**DOI:** 10.1055/a-2337-7551

**Published:** 2024-07-03

**Authors:** Jialiang Huang, Qinkai Li, Wei Wu

**Affiliations:** 1 105860 Gastroenterology, Second Affiliated Hospital of Soochow University, Suzhou, China

**Fig. 1:**
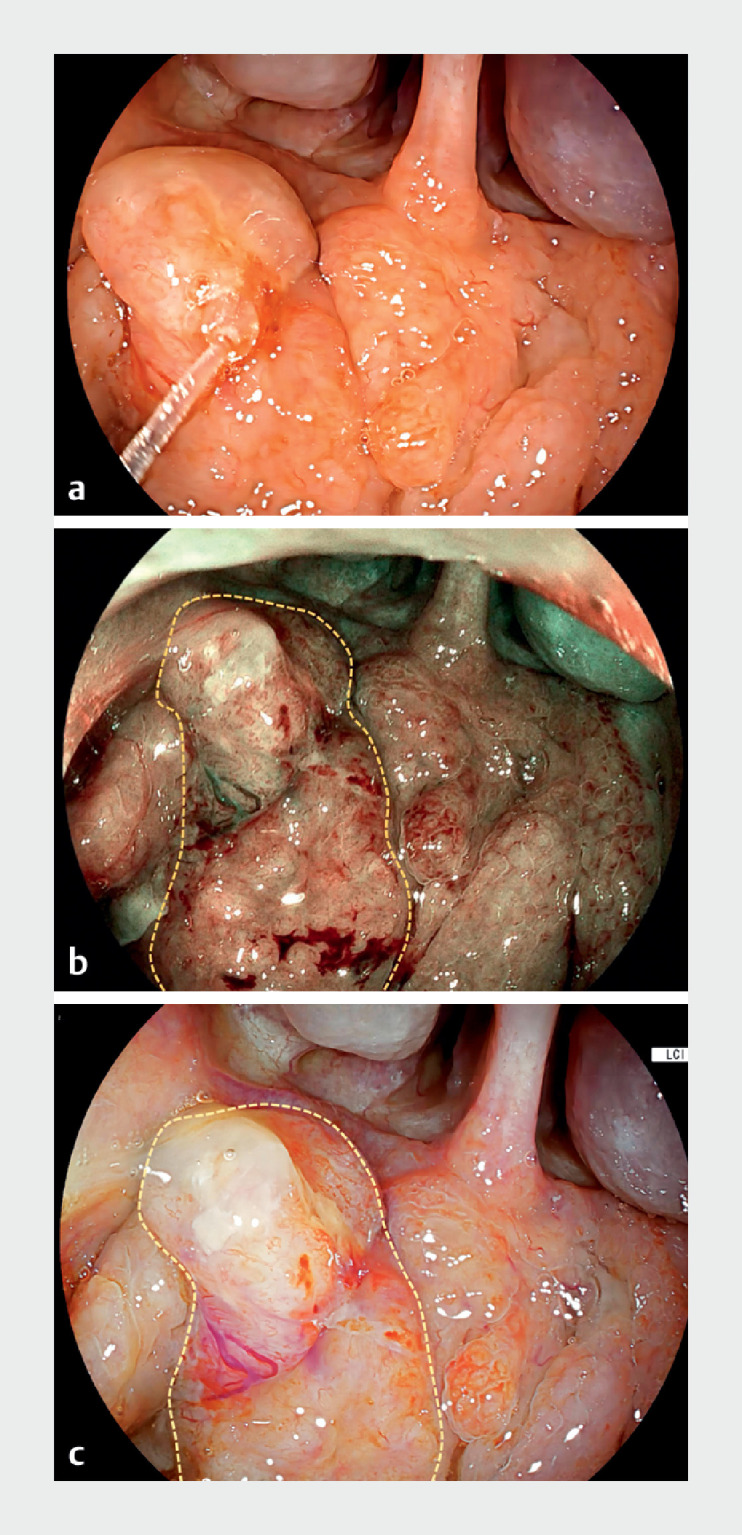
**Fig. 1a** Gastroscopic observation of squamous cell nonkeratinized carcinoma in the nasopharynx of a 45-year-old woman (dashed lines outline the lesion): **a** white light imaging; **b** blue light imaging; **c** linked color imaging.

**Fig. 2:**
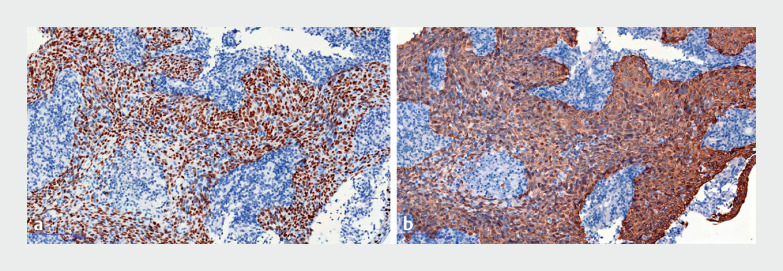
**Fig. 2a** Positive immunohistochemical staining: **a** P40 (× 100); **b** CK5/6 (× 100).

A 45-year-old woman presented at our hospital with epigastric pain. Gastroscopy was undertaken, but before the gastroscope was inserted into her esophagus, her nasopharynx was examined. This examination revealed a 1.8-cm lesion ( Video 1Video 1 ) near the pharyngeal opening of the eustachian (pharyngotympanic) tube on the right side. Blue light imaging and linked color imaging showed irregular microvasculature on the surface of the lesion ( Fig. 1Fig. 1 ). Pathological analysis identified squamous cell nonkeratinized carcinoma, with positive immunohistochemistry for P40 ( Fig. 2Fig. 2 **a** ) and CK5/6 ( Fig. 2Fig. 2 **b** ). The Ki-67 index was 50%. Subsequently, the patient was diagnosed with nasopharyngeal carcinoma. This is the first report of opportunistic screening for nasopharyngeal carcinoma using gastroscopy.

Observation of the nasopharynx by gastroscopy.Video 1Video 1

The nasopharynx is easy to access using a gastroscope. The gastroscope provides sufficient illumination and a high-quality visual field, and is equipped with electronic staining and magnification functions, which contribute to early detection of changes in the nasopharyngeal mucosal surface. It is particularly sensitive for detecting surface roughness, localized hyperplasia, and color changes, and enables any nasopharyngeal lesions identified to be biopsied. Opportunistic screening can enhance the detection rate of nasopharyngeal carcinoma, particularly in regions with a high incidence of this cancer.

Endoscopy_UCTN_Code_TTT_1AO_2AB

